# A patient choice‐driven lifestyle intervention lowers HbA1c in type 2 diabetes: A feasibility study

**DOI:** 10.14814/phy2.70163

**Published:** 2025-01-15

**Authors:** Nathan R. Weeldreyer, Mindy L. McEntee, Matthew P. Martin, Chong D. Lee, Farshad Fani Marvasti, Glenn A. Gaesser, Rodger Kessler, Siddhartha S. Angadi

**Affiliations:** ^1^ Department of Kinesiology, School of Education and Human Development University of Virginia Charlottesville Virginia USA; ^2^ College of Health Solutions Arizona State University Phoenix Arizona USA; ^3^ College of Medicine University of Arizona Phoenix Arizona USA; ^4^ Department of Family Medicine University of Colorado School of Medicine Denver Colorado USA

**Keywords:** exercise, HbA1c, nutrition, type 2 diabetes

## Abstract

Type 2 diabetes (T2D) is a common metabolic disorder in which only 25% of patients meet management targets. While the primary care setting is positioned to provide lifestyle management education, studies are lacking which integrate behavior interventions in this setting utilizing clinic staff. Thus, we evaluated a 90‐day lifestyle intervention for management of glycemia at a family practice clinic administered by clinic medical assistants. Twenty patients with non‐insulin‐dependent T2D completed a 90‐day intervention driven by patient choices of nutrition and physical activity. Medical assistants were trained by members of the study team and administered the intervention under nurse practitioner supervision. HbA1c trended toward significant reduction 8.59 ± 0.9% to 8.15 ± 1.2% (*p* = 0.051, 95% CI: −0.88 to 0.003). Modest reductions were observed for waist circumference (115.5 ± 12.6 vs. 112.5 ± 15.2 cm; *p* = 0.014, 95% CI: −5.66 to −0.26), body weight (97.7 ± 21.9 vs. 95.6 ± 23.9 kg; *p* = 0.016. 95% CI: −3.84 to −0.31), and BMI (33.7 ± 7.2 vs. 32.8 ± 7.5 kg/m^2^; *p* = 0.028, 95% CI: −1.29 to −0.12). This 90‐day, patient choice‐intervention was successful at lowering HbA1c in patients with T2D. Our study is limited by a lack of control group, and results should be interpreted as such. These data have implications for team‐based care models in clinic settings to improve health outcomes in patients with T2D.

## 1 INTRODUCTION

Type 2 diabetes (T2D) is a common metabolic disorder that affects 9.4% of the US population (Cannon et al., 2018). It is characterized by dysregulated lipid and carbohydrate metabolism resulting in hyperglycemia and insulin resistance (ADA, 2018; DeFronzo et al., 2015). These metabolic alterations lead to both microvascular and macrovascular damage and place patients with T2D at higher risk of cardiovascular disease (CVD) mortality. Indeed, CVD is the leading cause of death in this population (Prandi et al., 2022). However, disease control is poor with only 25% of diagnosed patients meeting glycemic, blood pressure, and lipid control targets (Kazemian et al., 2019).

In addition, the high prevalence of comorbid behavioral (such as depression and anxiety) and other social determinants of health seen in this population often contributes to poorer health outcomes and higher health care costs (Ducat et al., 2014; Haire‐Joshu & Hill‐Briggs, 2019; Kathol et al., 2014). Healthy lifestyle practices are valuable adjuncts to pharmacological therapy in patients with T2D. However, implementation of these can be resource intensive and cost‐prohibitive in family practice settings where clinician time is limited, and behavioral health expertise is lacking (Kathol et al., 2014; Knowler et al., 2002). Studies examining the effect of lifestyle interventions in T2D have often implemented a “one size fits all” approach in which all patients are given the same intervention. It is now recognized that giving patients increased say in their care and empowering them through diabetes education may lead to better outcomes (Heisler et al., 2021). The American Diabetes Association recommends this be administered by multidiscipline, team‐based care models to improve all health outcomes (American Diabetes Association Professional Practice Committee, 2022).

Thus, while the primary care setting is well positioned to offer population‐based management of T2D, lifestyle change‐based interventions have yet to be systemically evaluated or effectively integrated into clinic workflows. This pilot study examined feasibility and effectiveness of a behavioral, patient‐choice driven lifestyle intervention, delivered by trained medical assistants (MA) under the supervision of a nurse practitioner (NP), on hemoglobin A1c (HbA1c) in patients with T2D.

## 2 MATERIALS AND METHODS

### 2.1 Design

This brief patient‐centered intervention was designed around healthy lifestyle choices regarding nutrition and physical activity. Patients were instructed to select one nutrition and one physical activity option from a daily “à la carte” menu based on existing literature. Nutrition options included: (1) 8 tablespoons apple cider vinegar (Sprouts Brand, Phoenix, AZ), (2) 6 tablespoons psyllium husk powder (Sprouts Brand, Phoenix, AZ), (3) 60 g unsalted tree nuts, (4) 1 avocado, or (5) 6 tablespoons extra virgin olive oil (Sprouts Brand, Phoenix, AZ). Patients had flexibility in how they consumed the nutrition selections during the day. Patients were also given handouts educating them on how to read food labels and how to avoid refined sugars and replace them with complex carbohydrates. The exercise options were designed with the goal of meeting the American College of Sports Medicine's physical activity guidelines of at least 150 min of moderate‐intensity or 75 min of vigorous‐intensity activity per week (American College of Sports Medicine et al., 2021). Exercise options focused on walking and included: (1) three, 10‐min sessions per day, (2) one 30‐min session in the afternoon, (3) 2 min of exercise every hour at a moderate (60% heart rate max) or vigorous intensity (80% heart rate max), and (4) ten, 1‐min efforts at a high intensity throughout the day. Patients were given accelerometers (Fitbit Alta HR) to measure step counts and guide intensity based on predicted max heart rate (Tanaka equation) (Tanaka et al., 2001). Patients were not instructed to use specific exercise modalities to meet exercise goals. They were simply instructed to accumulate the desired time at each intensity however it fit their schedule and with the modality of their choice.

Two clinician–scientist authors (MPM and MLM) developed the behavioral support role for MAs, drawing from three evidence‐based approaches for health behavior change—motivational interviewing (Rotgers, 1993), Acceptance and Commitment Therapy (Hayes et al., 2012), and Problem‐Solving Therapy (D'zurilla & Goldfried, 1971). Clinic MAs received 10 h of 1:1 training from these authors in delivering protocol‐driven coaching sessions addressing participant motivation and problem‐solving barriers to diabetes self‐management.

### 2.2 Participants

A convenience sample of adults aged 30–80 being treated for T2D in a family medicine clinic participated in the brief 90‐day intervention. All aspects of the study were administered by clinic MAs and supervised by a family practice NP. Inclusion criteria were a diagnosis of T2D with an A1c of 7.5%–10%. Patients were excluded if they were on injectable insulin, had uncontrolled hypertension, were pregnant, or had any contraindications to exercise, or any allergies to foods included in nutritional goals. The study was conducted from 2019 to early 2020 and ethical approval was given by the Dignity Health Research Institute Arizona institutional review board. All participants provided written informed consent prior to enrollment in the study.

### 2.3 Procedures

Patients arrived at the clinic following an overnight fast (>8 h) test at baseline and post‐intervention. MAs measured participant height, weight, and waist circumference, then instructed patients to rest in a seated position for 15 min before central and brachial blood pressures were obtained (Oscar 2 with SphygmoCor, SunTech Medical). Fasted blood draws were performed via antecubital vein for measurement of glucose, lipids, and HbA1c and analyzed in the clinical labs at the family health clinic. MAs provided a total of six health coaching sessions throughout the intervention: three during office visits, three over the phone. In‐person visits occurred at baseline, 30, and 60 days and calls were given in between clinic visits. MAs asked open ended questions to address perceived barriers, motivating factors, and encourage patient engagement to help bolster adherence to the lifestyle intervention. Patients received $20 cash at each of the three required office visits along with a $50 grocery gift card that was to be used to help purchase the nutrition options. Study handouts can be viewed in the Figures S1–S3.

### 2.4 Statistical analyses

Because this was a feasibility study, only patients who completed the intervention were included in the analyses. All statistical tests were performed using SPSS statistical software (version 28). Patient change scores were calculated for each outcome measure and assessed for normality using Shapiro‐Wilks analysis. Based on these analyses, variables that were normally distributed were analyzed with paired‐samples *t*‐tests. Those that were nonparametric were analyzed with Wilcoxon‐Signed Rank tests. In order to examine the potential role of sex, independent samples *t*‐tests were run on all outcome variables. Pearson's (*r*) or Spearman's correlations (*ρ*) were used to look for relationships between changes in body composition and metabolic blood markers. Cohen's *d* effect sizes were calculated to examine magnitude of changes due to the nature of the study design. Data are reported as mean ± SD.

## 3 RESULTS

Fifty‐one patients were consented and screened for this study. Seventeen of these were screen failures, predominantly due to HbA1c not meeting inclusion criteria. Thirty‐four patients began the intervention, three withdrew from the trial and 11 were lost to follow up. Twenty patients met inclusion criteria and completed all study visits. Baseline characteristics can be found in Table 1.

**TABLE 1:** Baseline characteristics.

Characteristic	Patients (*n* = 20)
Age (years)	56.7 ± 12
Sex	
Male	9
Female	11
Ethnicity	
Caucasian	12
African American	6
Latino	1
Preferred not to answer	1
BMI, kg/m^2^	33.7 ± 7.1
HbA1c, %	8.6 ± 0.9
Type II diabetes duration, years	8.25
Medications, *n* (%)	
Metformin	19 (95%)
GLP‐1a	0 (0%)
Sulfonylureas	8 (40%)
SGLT‐2i	2 (10%)
DPP‐4i	4 (20%)
Statin	12 (60%)
Beta‐blocker	2 (10%)
Ace‐inhibitor	9 (45%)
Diuretics	6 (30%)

*Note*: Data presented as mean ± SD.

**FIGURE 1:**
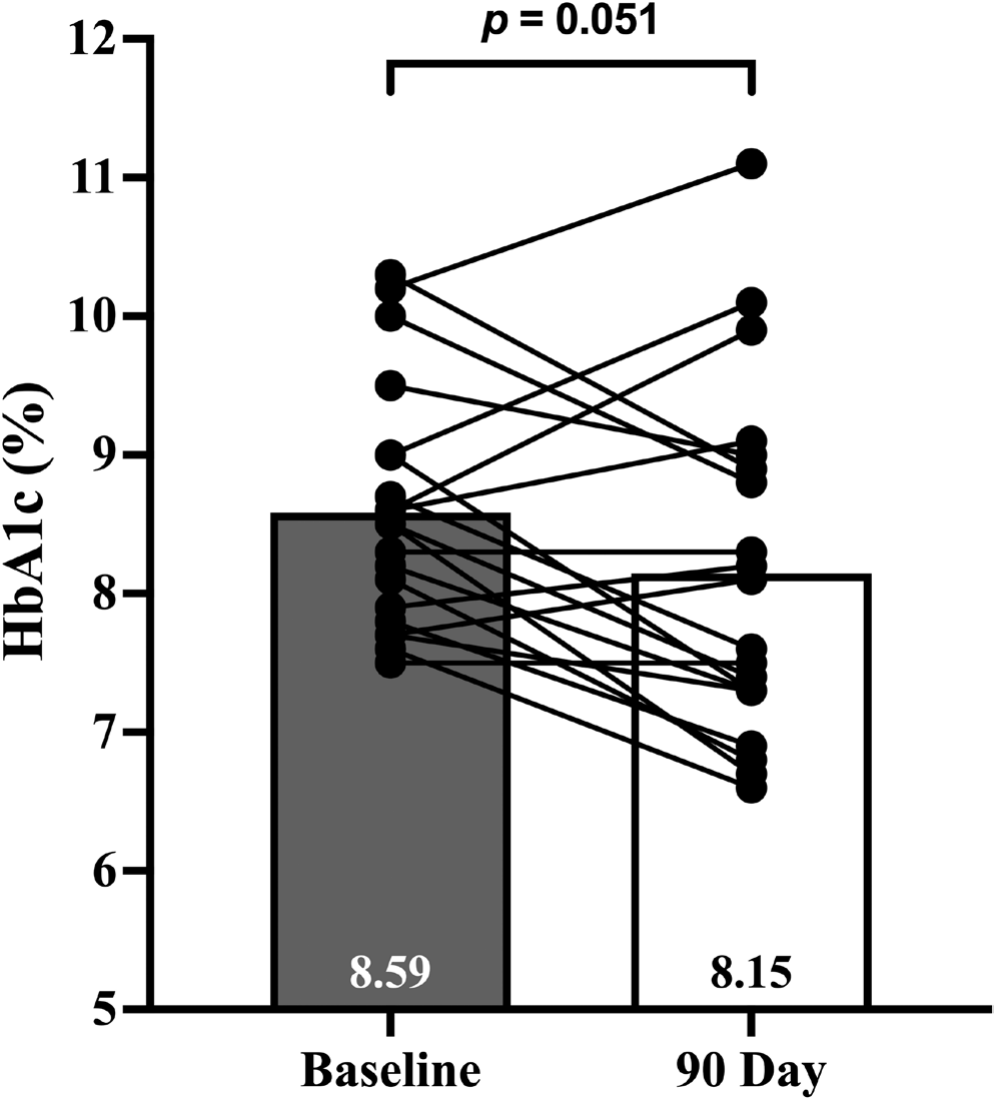
Mean and individual changes in HbA1c (%).

The mean reduction in HbA1c was 0.44% from pre‐ to post‐testing (8.59 ± 0.9 vs. 8.15 ± 1.2%; *p* = 0.051; 95% CI: −0.88 to 0.003) (Figure 1). Additionally, modest reductions occurred for waist circumference (115.5 ± 12.6 vs. 112.5 ± 15.2 cm; *p* = 0.014; 95% CI: −5.66 to −0.26), body weight (97.7 ± 21.9 vs. 95.6 ± 23.9 kg; *p* = 0.016; 95% CI: −3.84 to −0.31), and BMI (33.7 ± 7.2 vs. 32.8 ± 7.5 kg/m^2^; *p* = 0.028; 95% CI: −1.29 to −0.12). Brachial and central blood pressures, fasting glucose, insulin, and lipids were not changed (Table 2). No significant effect of sex was found for any outcome measure (all *p* > 0.1).

**TABLE 2:** Anthropometry, blood pressures, and clinical biomarkers at baseline and 90‐day visits.

	Baseline	90 Day	∆ (95% CI)	*p*	d
Anthropometry					
Weight, kg	97.7 ± 21.9	95.6 ± 23.9	−2.1 (−3.84 to −0.31)	0.024*	0.55
BMI, kg/m^2^ ^a^	33.7 ± 7.2	32.9 ± 7.5	−0.8 (−1.29 to −0.12)	0.028*	0.49
Waist circumference, cm	115.5 ± 12.6	112.5 ± 15.2	−3.0 (−5.66 to −0.26)	0.033*	0.51
Hemodynamics					
SBP, mmHg	135 ± 19	131 ± 18	−4 (−12.68 to 3.86)	0.28	0.25
DBP, mmHg	80 ± 13	77 ± 10	−3 (−7.71 to 1.15)	0.14	0.35
cSBP, mmHg	127 ± 17	123 ± 17	−4 (−10.42 to 4.71)	0.44	0.19
cDBP, mmHg	83 ± 14	80 ± 7	−3 (−9.62 to 1.19)	0.12	0.40
Aix, %	42.7 ± 9.7	44.0 ± 13.5	1.3 (−7.36 to 8.34)	0.90	0.03
Blood markers					
HbA1c, % (mmol/mol)	8.59 ± 0.9 (70)	8.15 ± 1.3 (66)	−0.44 (−0.88 to 0.003)	0.051	0.47
Fasting glucose, mg/dl	190 ± 43	186 ± 84	−4 (−31.87 to 23.97)	0.78	0.06
Fasting insulin mlU/L^a^	23.7 ± 12.9	26.9 ± 23.9	3.2 (−8.50 to 4.90)	0.327	0.21
HOMA‐IR^a^	11 ± 5	12 ± 16	1 (−5.1 to 2.25)	0.349	0.21
Total cholesterol, mg/dL	169 ± 56	166 ± 45	−3 (−15.58 to 8.48)	0.55	0.13
LDL, mg/dL^a^	92 ± 46	89 ± 43	−3 (−8.00 to 8.50)	0.940	0.02
HDL, mg/dL	44 ± 10	45 ± 10	1 (−1.71 to 4.21)	0.39	0.20
Triglycerides, mg/dL^a^	168 ± 71	158 ± 72	−10 (−32.5 to 17.5)	1.00	0.00

Abbreviations: Aix, augmentation index (%); cDBP, central diastolic blood pressure; cSBP, central systolic blood pressure; DBP, diastolic blood pressure; SBP, systolic blood pressure.

^a^ Represents variables which are non‐normally distributed and were assessed via Wilcoxon Signed Ranks test. Data presented as mean ± SD.

*p ≤ 0.05.

**FIGURE 2:**
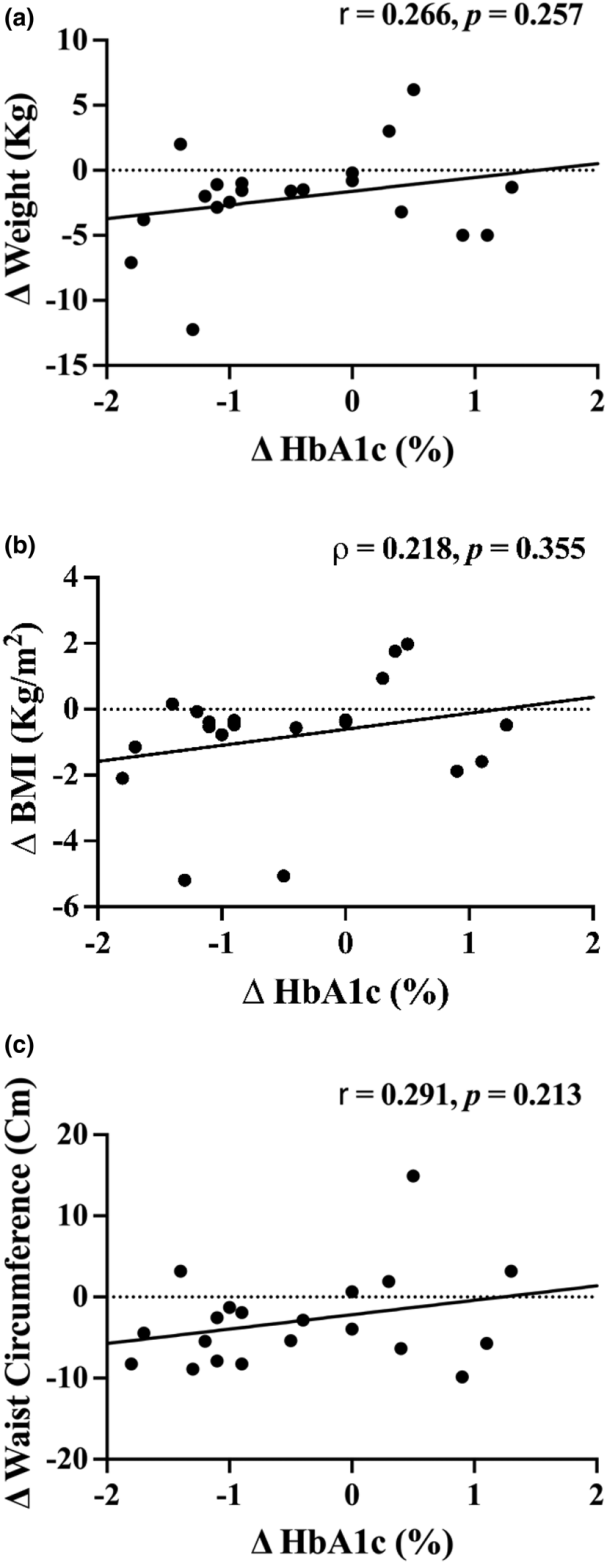
Correlations between changes in HbA1c and anthropometric measures.

Changes in HbA1c were not correlated with changes in body weight (*r* = 0.252, *p* = 0.283), BMI (*ρ* = 0.218, *p* = 0.355) or waist circumference (*r* = 0.218, *p* = 0.356) (Figure 2.). Although mean serum triglyceride concentration was not significantly decreased during the intervention, changes in serum triglyceride concentration were moderately correlated with changes in body weight (*ρ* = 0.539, *p* = 0.014) and waist circumference (*ρ* = 0.494, *p* = 0.027).

## 4 DISCUSSION

The primary outcome of our study was to evaluate the change in HbA1c during the intervention. The main findings from this study were that our 90‐day, patient‐choice driven intervention showed clinically significant reductions in HbA1c (Inzucchi et al., 2018; Sacks et al., 2024). These findings may have important clinical implications as the increased glycemia associated with this is largely implicated in the micro and macrovascular complications and increased CVD risk associated with T2D (Forbes & Cooper, 2013). Modest reductions were also observed for body weight, BMI, and waist circumference, although these changes were not associated with reduced HbA1c. No significant reductions were observed for fasting glucose, insulin, lipids, or blood pressure. However, both BP and lipids were well‐controlled at baseline and thus, it's plausible that this was due to a ceiling effect. Our results suggest that training and using clinic MAs to administer a patient choice‐driven lifestyle intervention under the supervision of a nurse practitioner may be an effective strategy for reducing HbA1c in an outpatient setting.

While patients were instructed to remain medication stable throughout the study intervention, four patients did have changes made to glucose‐lowering medications. One patient had their prescription to Metformin removed due to improved glucose levels and caution for hypoglycemia. They had a reduction in HbA1c of 1.2%. Another had Metformin added to their prescription; however, no change in HbA1c was seen in this patient. A third was started on Glyburide during the last 30 days of the study period. Finally, one patient was started on insulin the week of post testing due to worsening glycemia (an increase in HbA1c of 0.5% was seen).

It is important to note that there were no significant relationships seen between changes in HbA1c and body weight, BMI, or waist circumference, a commonly used metric to estimate visceral adiposity. While our study is underpowered and risk of type II error exists, this finding further adds to the body of literature showing that weight loss alone does not lead to improved health outcomes (Gaesser & Angadi, 2021). Indeed, exercise training in patients with T2D can lead to significant reductions in HbA1c (−0.66%) while no significant differences are seen in body weight between exercisers and controls (Boule et al., 2001). In addition, a meta‐analysis by Snowling and Hopkins found 3 times higher weight loss during combination training but both aerobic and combination training showed similar HbA1c reductions (0.7% and 0.8%, respectively) (Snowling & Hopkins, 2006) indicating no clear relationship between weight loss and improved glycemia.

There is an increasing body of evidence that utilizing clinical staff, such as MAs, for interventions may lead to enhanced patient outcomes following adequate training (Heisler et al., 2021). In this study, MAs received a total of 10 h of training from study physicians, behavioral health clinicians, dieticians, and exercise physiologists in which they were instructed on how to conduct all study procedures. Following this training, they were able to successfully implement the behavioral health coaching intervention and all aspects of the study.

Studies have previously utilized MAs due to their being more aligned with patient populations and their financial advantages (i.e., lower billing costs) (Willard‐Grace et al., 2015). However, results from these studies have been largely unsuccessful at reducing clinically relevant biomarkers (Buhse et al., 2018; Ruggiero et al., 2010, 2014; Willard‐Grace et al., 2015). This may be due in part to these studies often only implementing behavioral health coaching. While the current study also implemented a behavioral health component, patients were provided with a menu giving specific nutrition and exercise prescriptions to follow. In addition, this dietary modification and exercise intervention allowed for a greater agency from patients as it allowed for daily choices to be made to suit their lifestyles and preferences. This may improve patient adherence and long‐term engagement in healthy lifestyle choices (Bluml et al., 2019; Heisler et al., 2021).

Due to the large healthcare burden of diabetes, numerous lifestyle interventions have been utilized to improve patient outcomes. Programs such as the Diabetes Prevention Program are effective at improving glycemic control but are time‐ and resource‐intensive due to their supervised nature (Knowler et al., 2002). A meta‐analysis from Snowling and Hopkins showed that aerobic, resistance, or combination exercise interventions demonstrated a mean HbA1c reduction of 0.8% for studies with durations of ≥12 weeks or 0.4% if <12 weeks (Snowling & Hopkins, 2006). This was at the time–cost of 58 ± 44 h per subject for the interventions included, which would be difficult to implement in a primary care setting. However, we demonstrated a 0.44% reduction in HbA1c with substantially lower time utilization since our study was designed to minimize clinic burden. In fact, MAs on this study spent ~7 h per subject for visits and phone calls over the 3‐month intervention (an ~8‐fold lower time burden).

This study is not without limitations. First, only 20 subjects from a single clinic completed pre‐ and post‐data included in analysis. Our sample collection was cut short due to the COVID‐19 pandemic which caused early study termination. Additionally, there was no control group. As a result, we cannot definitively say changes in outcomes measures are fully atrribuatable to the intervention prescribed. Finally, the study duration was only 3 months. Future studies should address these limitations by including a comparative control group as well as examining if a longer intervention could lead to further improvements in HbA1c or maintained reductions from baseline.

## 5 CONCLUSION

In conclusion, this 90‐day clinic based, patient‐choice driven lifestyle intervention administered by medical assistants in a primary care setting was effective for reducing HbA1c, body weight, BMI, and waist circumference in patients with type 2 diabetes. Results suggest behavioral support from non‐licensed staff, such as MAs or community health workers, may serve as an effective strategy for improving chronic diabetes care in clinic settings.

## AUTHOR CONTRIBUTIONS

N.R.W. assisted with data collection, performed the statistical analysis, and drafted the manuscript. S.S.A., G.A.G., and R.K. were responsible for conceptualization and methodology. S.S.A. and R.K. were responsible for funding acquisition. M.L.M. and M.P.M. developed the behavioral intervention and trained clinic MAs in administration. C.D.L. served as study statistician. All authors reviewed, approved the manuscript, and consented to its publication.

## FUNDING INFORMATION

This study was supported by the Dignity Health and Arizona State University Collaborative Strategic Initiatives Program.

## CONFLICT OF INTEREST STATEMENT

The authors declare that the research was conducted in the absence of any commercial or financial relationships that could be construed as a potential conflict of interest.

## ETHICS STATEMENT

Ethical approval was given by the Dignity Health Research Institute Arizona institutional review board. All participants provided written informed consent prior to enrollment in the study.

## Supporting information


Figures S1–S3.

## Data Availability

Data from this study will be made available upon reasonable request.
