# Supplementary material for: A patient choice‐driven lifestyle intervention lowers HbA1c in type 2 diabetes: A feasibility study
Source: Physiol Rep. 2025 Jan 15;13(2):e70163. doi: 10.14814/phy2.70163 (PMC11735460; doi:10.14814/phy2.70163)

**Supplementary Figure 1.**

Intervention menu given to subjects. Subjects were instructed to select 1 option from both the nutrition and exercise sides of the menu each day.


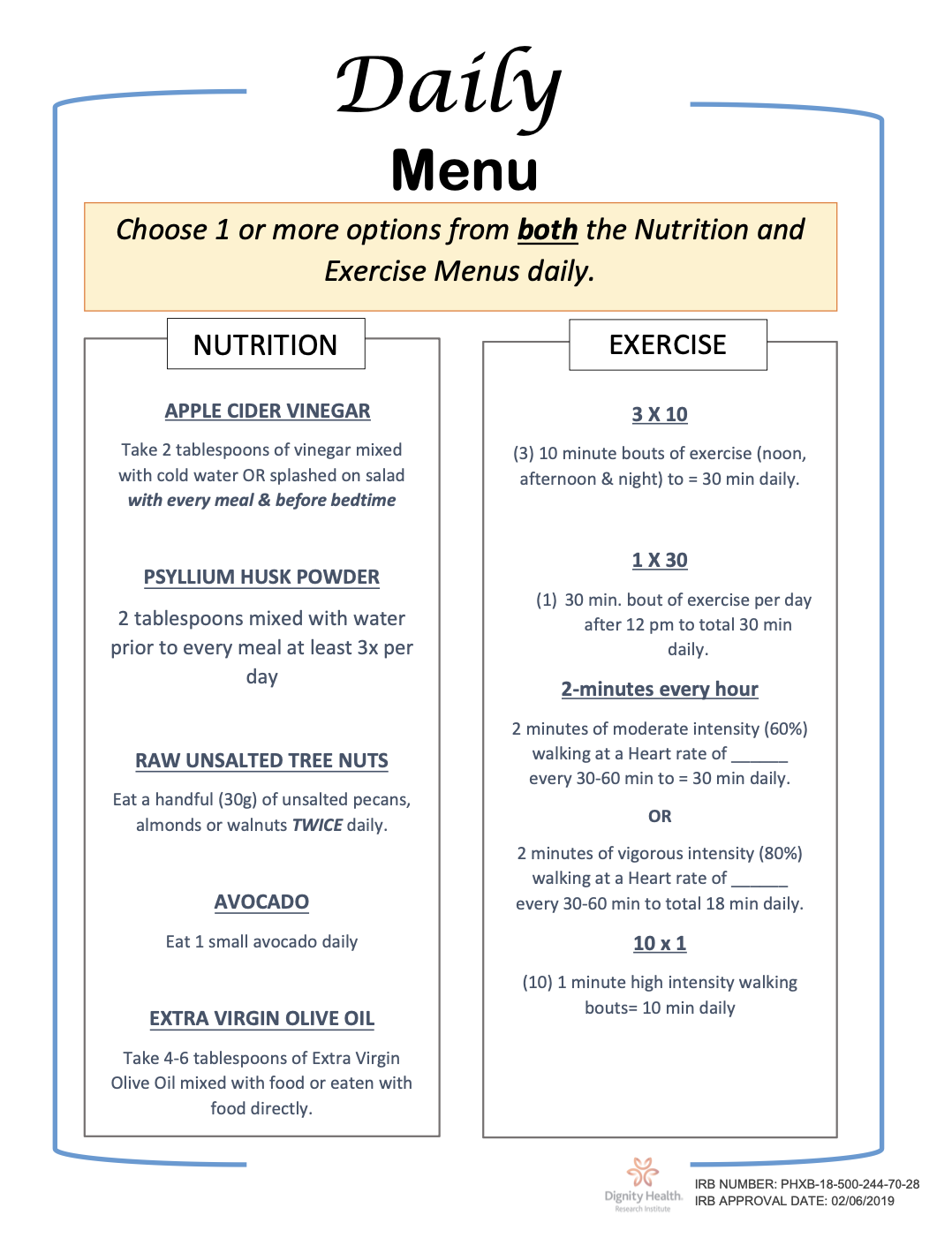


**Supplementary Figure 2.**

Nutritional education handouts given to subjects. These included tips to implement choices from the daily menu as well as foods to avoid, and instructions on how to read food labels.


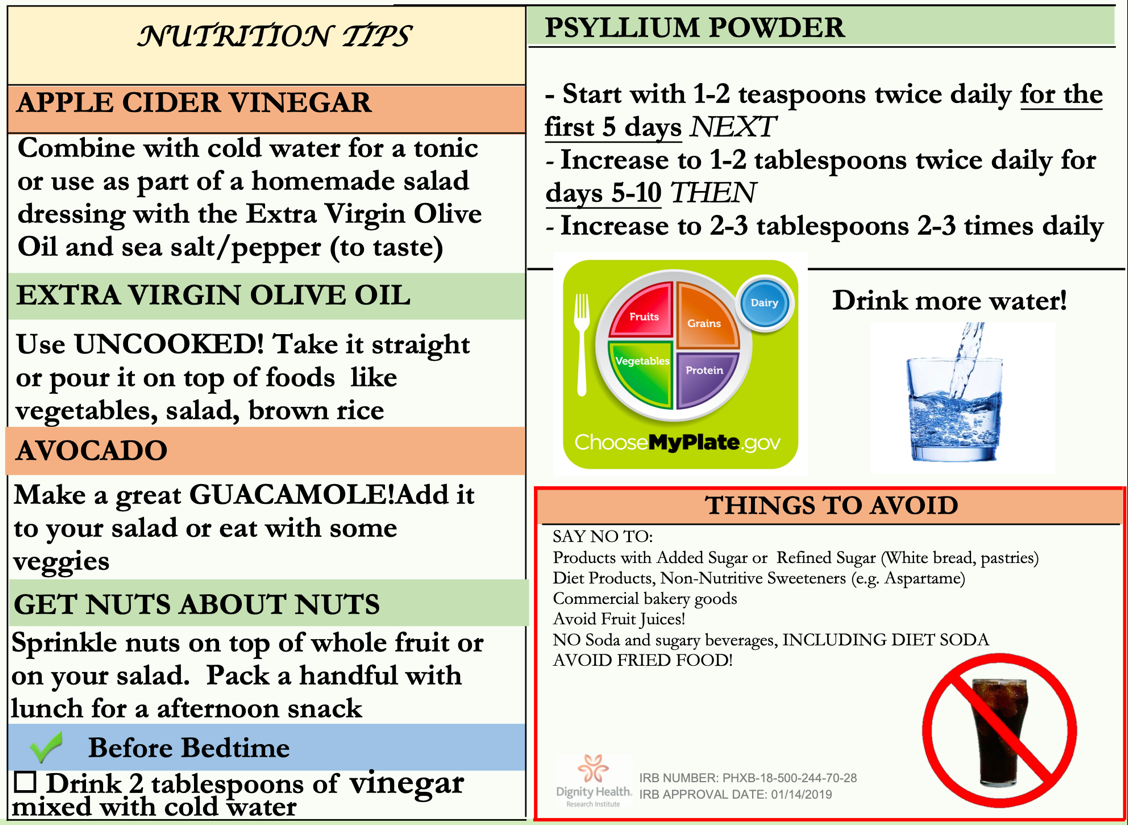


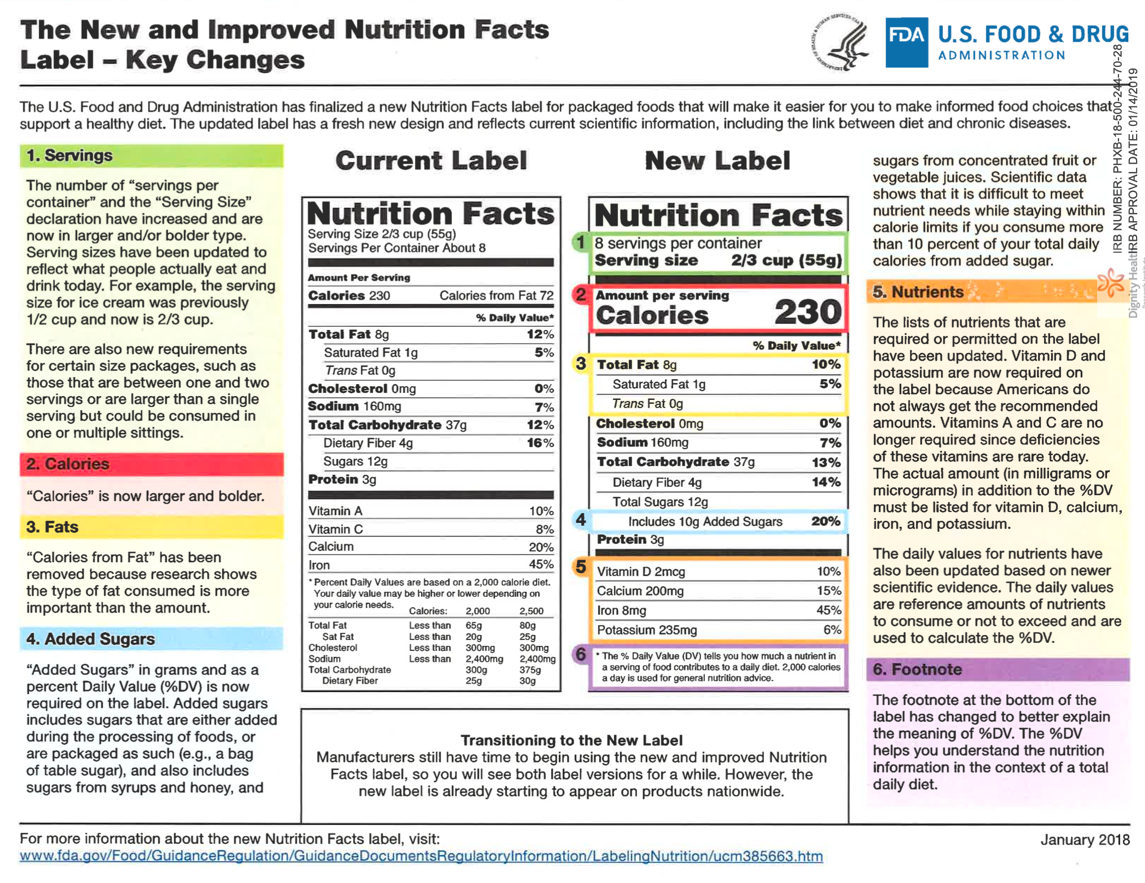


**Supplementary Fig. 3**

Additional educational handouts given to patients on how to choose and prepare foods.


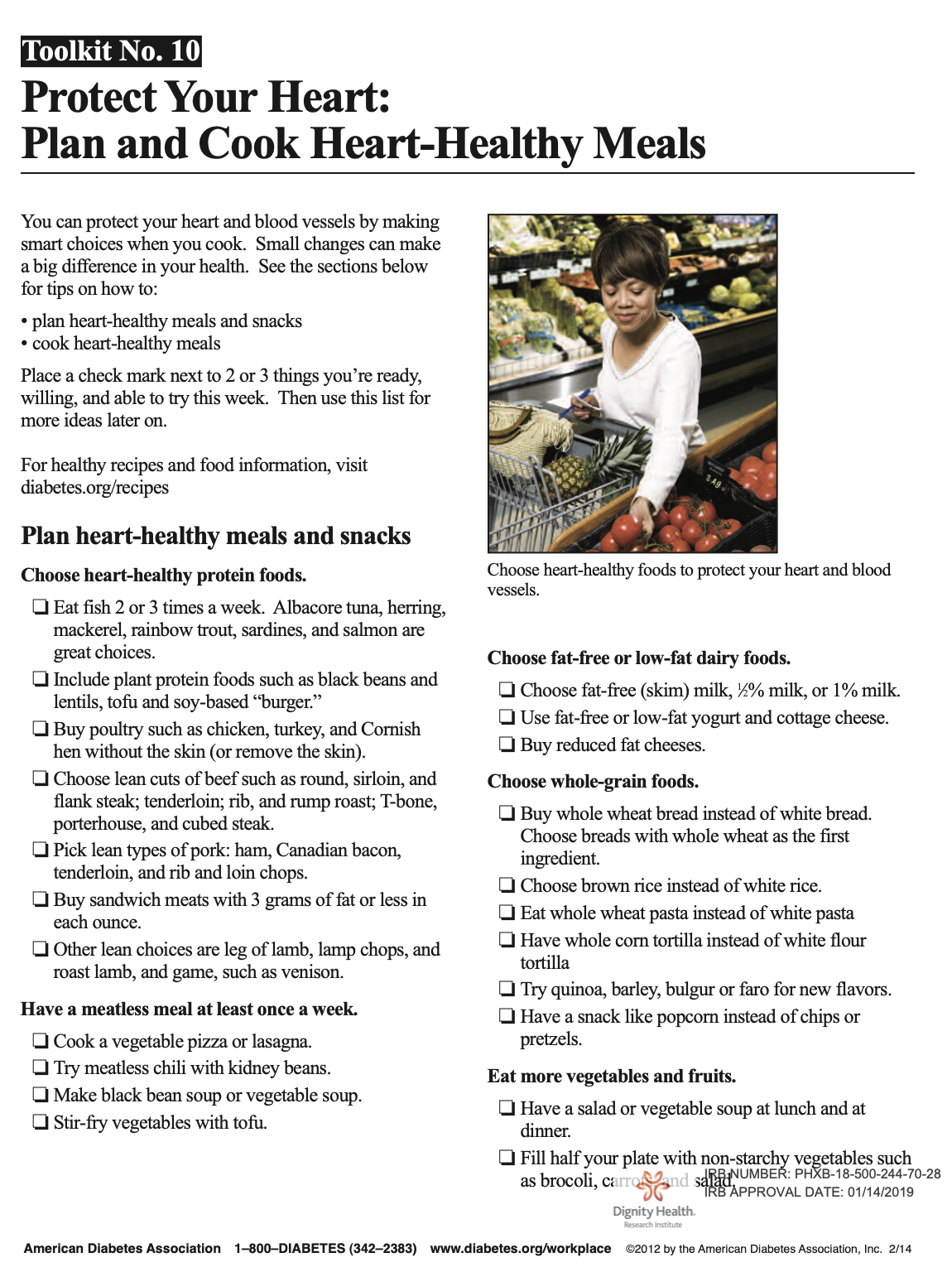


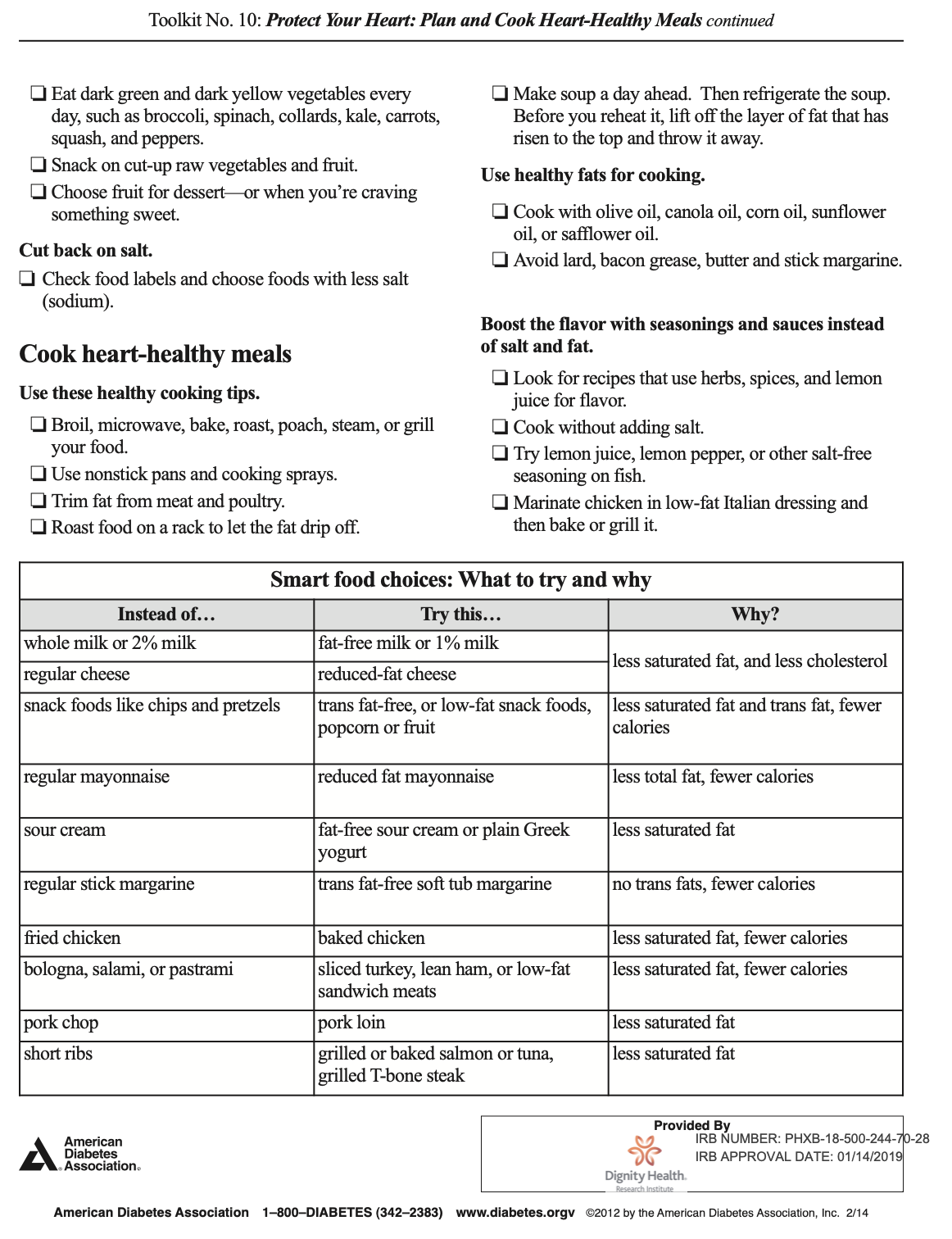

Supplement: Supplementary file 1 — Figures S1–S3. [file PHY2-13-e70163-s001.docx]
